# “Assessing inclusiveness in Team Europe Initiatives: a mixed-methods study of EU-Africa health cooperation”

**DOI:** 10.1080/16549716.2026.2680837

**Published:** 2026-06-03

**Authors:** Saskia-Linda Stämmler, Nina Viberg, Louise Bengtsson

**Affiliations:** a Department of Global Public Health, Karolinska Insitutet, Stockholm, Sweden; b Department of Global Governance, Regulation, Innovation and Digital Economy (GRID), Centre for European Policy Studies, Brussels, Belgium

**Keywords:** Development cooperation, equitable partnerships, global health governance, stakeholder perspectives, power dynamics

## Abstract

**Background:**

Team Europe Initiatives have become a central mechanism for the European Union’s engagement as a global development actor. Grounded in principles of inclusive partnership, the initiatives aim to improve the effectiveness and visibility of European contributions. However, questions remain about the geographical inclusiveness of health-related initiatives and the extent to which they incorporate local priorities and actors.

**Objectives:**

This study examines how health-related Team Europe Initiatives are distributed geographically and explores stakeholder perceptions of their inclusiveness, partnership dynamics, and implementation on the ground.

**Methods:**

A mixed-methods design was used, combining a quantitative mapping of the distribution of health-related Team Europe Initiatives with 23 semi-structured interviews conducted between January and April 2024 with stakeholders in Europe and Africa. Quantitative findings informed the qualitative sampling and analysis. The interviews were analysed using thematic analysis.

**Results:**

Health-related Team Europe Initiatives are predominantly concentrated in Africa, with uneven participation across European Union Member States. Interviewees generally viewed the initiatives as a promising tool for strengthening collaboration, improving trust, enhancing coordination, and creating more stable engagement that may attract investment. Nonetheless, significant concerns persist, particularly regarding limited involvement of local partners in decision-making, resulting power imbalances, and challenges when it comes to coordination. Stakeholders broadly agreed on the need to widen participation beyond European institutions to meaningfully reflect local priorities.

**Conclusions:**

While Team Europe Initiatives hold potential to support more inclusive and coordinated global health action, the initiatives’ impact could be enhanced by strengthening the role of partner-country actors in their formulation and implementation.

## Paper context

- **Main findings:** Health-related Team Europe Initiatives are viewed as a promising tool for collaboration, but concerns persist regarding meaningful involvement of non-European partners.
- **Added knowledge:** This study provides real-world quantitative and qualitative insights into the geographical distribution and inclusiveness of health-focused Team Europe Initiatives.
- **Global health impact for policy and action:** Strengthening the role of local partners in decision-making and expanding participation beyond European actors may enhance the impact of Team Europe Initiatives.

## Background

In her role as President of the European Commission, Ursula von der Leyen has urged the European Union (EU) to strengthen its ‘responsible global leadership’ and promote a values-driven external action agenda [1]. These principles have increasingly embraced inclusivity, equitable partnership, and local ownership [2]. However, recent developments suggest a shift in the EU’s external engagement, particularly in health and development cooperation, toward a more interest-driven and geopolitical orientation [3]. This turn also aligns with the global health resilience initiative, announced by von der Leyen in her 2025 State of the Union address.

This balancing between equitable partnerships and the ambition to establish the EU as a global health leader is noticeable in EU-Africa relations. Africa is perceived by the EU as a crucial development partner and a geopolitical player. The COVID-19 pandemic exposed vulnerabilities in global health systems, supply chains, and governance structures, prompting a re-evaluation of global partnerships [4]. These structural challenges have been interpreted by the European Commission as developments that call for a strategic, coherent, and inclusive global health architecture for low- and middle-income countries. Against this backdrop, the EU introduced the Team Europe approach in 2020 as a coordinated response to the pandemic [5]. Team Europe aimed to streamline EU and Member State (MS) efforts by pooling resources, aligning objectives, and global visibility. Over €53 billion was mobilized under this model to support international partners [6]. Since then, Team Europe Initiatives (TEIs) have become the EU’s primary governance model for external development cooperation, extending into areas such as digital transformation, green transition, and health.

TEIs represent a strategic pivot. They move beyond traditional donor relationships by leveraging the EU’s broader political and economic considerations. Ultimately, TEIs aim to strengthen the EU’s position as a credible and coordinated global actor [7]. Embedded in the EU’s Global Gateway strategy, TEIs are designed to foster inclusive and impact-oriented partnerships across sectors [8]. They involve a wide array of actors, including EU institutions and MS, financial institutions, civil society organizations (CSO), and partner countries. In practice, TEIs represent coordinated activities by different Team Europe actors under jointly agreed objectives, focusing on collective impact in specific areas or regions (Table 1). Despite their design, TEIs face notable implementation challenges. Studies have highlighted persistent coordination difficulties, unclear definitions of MS contributions, and a lack of transparency in operationalization [6,7]. An evaluation by the Swedish Expert Group for Aid Studies (EBA, 2022) highlighted insufficient financial and human resources at EU delegation overseas missions, managing development cooperation and representing the EU locally. Compared to centrally based institutions in Brussels, these delegations face staffing constraints, contributing to centralization that limits responsiveness and local relevance [9].

**Table 1.** Key elements of Team Europe Initiatives.

*Partizipation*	A TEI consist of several EU Member States, including their agencies and development banks, which contribute with either expertise or other resources. Participation in a particular TEIs is open to all MS, but at least four have to participate actively. The other members of a TEI are the European Union (typically the European Commission) as well as the European Investment Bank (EIB) and the European Bank for Reconstruction and Development (EBRD). Occasionally, non-Team Europe actors participate as partners.
*Flexibility and leverage*	TEIs aim to increase both flexibility and joint leverage in the EUs external activities, by pooling the resources and expertise of its members. As participation is voluntary, it also allows a group of actors to move ahead around a particular strategic priority, without all EU Member States having to agree on the approach.
*Strategic interests*	TEIs align with the EUs strategic interests and policy priorities as a global actor. These include the green transition, digitalization and peace and security and migration management. Partly but not fully, these align with the EU’s commitment to the SDGs and principles of development cooperation.

In addition to structural issues, thematic imbalances within the totality of the EU’s TEIs have also emerged. Despite the adoption of the EU Global Health Strategy in 2022, which reaffirmed health as a priority in EU development policy [10], most TEIs remain focused on economic, digital, or environmental domains. Health-related TEIs are few, and their design and implementation remain under-researched. This gap is particularly concerning given the centrality of health to the Sustainable Development Goals (SDG 3) and the lessons from the COVID-19 pandemic [11].

Inclusiveness represents another concern. Although TEIs promote inclusive, multi-stakeholder collaboration, the extent to which partners, particularly those in the Global South, are involved in agenda-setting and decision-making remains ambiguous. Participation often reflects EU strategic interests more than partner country priorities [12,13]. Moreover, local perspectives can be sidelined in favor of donor-driven priorities, risking undermining local ownership and sustainability [14].

Aligning development initiatives with national health strategies is widely recognized as key to strengthening country ownership and ensuring external support reflects local priorities [15]. In EU-Africa cooperation, this alignment may enable TEIs to support nationally defined health priorities while fostering inclusive partnerships between international and local actors.

This study examines the inclusiveness of health-related TEIs in EU – Africa cooperation. Using a mixed-methods approach combining quantitative mapping and qualitative interviews, it explores stakeholder participation, perception, and the influence of partner countries on TEI design and implementation. Addressing gaps in the literature, the study contributes to understanding how inclusive and responsive EU global health engagement is in practice.

## Methods

### Study design

This study adopts a mixed-method approach combining qualitative and quantitative analysis to examine inclusiveness in TEIs as both a structural characteristic and a socially negotiated process. Inclusiveness is operationalized as measurable indicators, such as geographic distribution, MS participation, formal involvement of local partners, perceptions of participation, influence, and power-sharing among stakeholders.

Inclusiveness is defined as the extent to which diverse stakeholders, particularly local actors, are engaged across all stages of the development cooperation process, encompassing the design, implementation, monitoring, and evaluation.

The conceptualization builds on international frameworks emphasizing equity, participation, accountability, and local ownership, notably the Busan Partnership for Effective Development Cooperation [15] and the EU Global Health Strategy [10]. Three analytical dimensions guide the assessment of TEIs:

1. Geographic Inclusiveness

This dimension refers to the distribution of TEIs and the extent to which they target diverse regions. It examines how geographic focus shapes local partnerships.

2. Actor Inclusiveness

This evaluates the diversity of stakeholders involved in TEIs beyond state actors. It assesses engagement with diverse actors and whether they are consulted or genuinely empowered.

3. Power and Decision-Making

This dimension investigates the balance of power between EU actors and local stakeholders. It examines who sets priorities, makes decisions, and allocates resources.

### Data collection and sampling

The study follows a two-phase sequential design mixed-method design, consisting of an initial quantitative mapping followed by a qualitative phase aimed at exploring stakeholder perceptions of inclusiveness [16]. In the first phase, data were extracted from the European Commission’s Joint Programming Tracker (January–February 2024) [17].

Twenty-three semi-structured interviews were conducted (January–April 2024), with stakeholders from both European and African contexts, including representatives from national governments, financial institutions, international organizations, and private sectors. To balance sampling bias, participants were deliberately selected based on the results of the mapping in the first phase (e.g. underrepresented groups in the TEIs, such as smaller EU MS, local actors in Africa, non-state actors involved). This diversity ensures perspectives from policymaking, implementation, development cooperation, and industry in the study. Table 2 provides information of interviews conducted.

**Table 2.** List of the 23 interviews conducted.

Interview ID	Geographically located (Office)	Represented Sector	Gender
Interview 1	Africa	International Organization	Female
Interview 2	Europe	European Commission Institution	Male
Interview 3	Europe	European Member State Agency	Male
Interview 4	Europe	European Member State Agency	Female
Interview 5	Africa	European Commission Institution	Female
Interview 6	Europe	European Member State Agency	Male
Interview 7	Africa	European Member State Institution	Female
Interview 8	Africa	African Governmental Institution	Male
Interview 9	Europe	European Member State Agency	Male
Interview 10	Africa	International Organization	Male
Interview 11	Europe	European Member State Agency	Female
Interview 12	Europe	European Member State Agency	Male
Interview 13	Europe	European Member State Agency	Female
Interview 14	Africa	European Member State Institution	Female
Interview 15	Europe	European Member State Agency	Female
Interview 16	Africa	International Organization	Female
Interview 17	Europe	Private Sector Company	Female
Interview 18	Europe	European Member State Institution	Female
Interview 19	Europe	European Commission Institution	Male
Interview 20	Africa	International Organization	Male
Interview 21	Africa	Private Sector Company	Male
Interview 22	Africa	European Member State Agency	Male
Interview 23	Africa	African Governmental Institution	Male

The study is grounded in a conceptual framework of inclusivity, which builds on critiques of TEIs, particularly their perceived lack of local ownership, stakeholder diversity, and power-sharing mechanisms [7,11,18].

A purposive sampling strategy was used to select individuals directly involved in health-related TEIs and development cooperation. To increase the diversity of perspectives, snowball sampling was used, as interviewees were highly interconnected, and public data from the first phase did not capture all stakeholders in Africa [19].

Participants were contacted via e-mail and provided both written and oral informed consent. Interviews were conducted online or in person. All interviews were recorded, transcribed, and anonymized.

The interview guide was developed based on recent literature on development cooperation and global health partnerships [20,21], with a specific emphasis on perceptions of inclusiveness in EU-led health initiatives and furthermore, with the help of professionals working in development cooperation. The guide was tested through two pilot interviews with researchers working in Africa. The guide is included under supplementary material. Table 3 summarizes TEIs covered by interviews.

**Table 3.** Example of the thematic analysis process, with generating codes, identifying themes and subthemes, and supporting the data with quotations from the interviews.

Name of Team Europe Initiative	Sectorial focus	Operational Level	Thematic priority and status
Manufacturing and Access to Vaccines, Medicines, and Health Technologies (MAV+)	Increasing equitable access to safe, effective, quality, and affordable essential vaccines, medicines, and health technologies for all.	Regional	Human Development Launched in 2021. In Implementation.
Digital Health- Africa	Digital for COVID − 19, digital tools, skills, and transformation in support to the COVID-19 response. Digitalization of Health systems. Strengthening and Universal Health Coverage (UHC).	Regional	Science, Technology, Innovation and Digital Human Development Launched in 2024. Development is ongoing.
Sexual and Reproductive Health and Rights in Sub-Saharan Africa (SRHR)	Advancing the SRHR agenda, with a particular focus on adolescent girls and young women	Regional	Human Development Launched in 2021. In Implementation.
Public Health Capacity- Africa	On a national level support services of schools and institutions of public health in terms of research, disease prevention and health promotion. Evaluation and promotion of equitable access to the services.	Regional	Technology, Innovation and Digital Human Development Launched in 2022. In Implementation.
Sustainable Health Security- Africa	To strengthen sustainable, risk-informed prevention, preparedness, and response to infectious threats and antimicrobial resistance (AMR)	Regional	Human Development Launched in 2022. In Implementation.

### Data analysis

Quantitative data was analyzed using descriptive statistics to visualize the geographical and thematic distribution of TEIs, using STATA 18.0 [22]. Key variables included the number of TEIs per region, MS participation, and areas of health focus. Findings informed regions and participants for the qualitative phase.

The qualitative data was analyzed using reflexive thematic analysis with an inductive approach [23]. Open coding was applied to identify emerging themes related to the inclusiveness of TEIs. Coding was conducted manually by the lead researcher, and an example of the coding framework is presented in Table 4. Integration of both data sources was carried out at the interpretation stage of the analysis. This was reflected by all authors. Quantitative findings provided data on the distribution, scope, and thematic focus of health-related TEIs, while qualitative findings offered insights into how inclusiveness was perceived by stakeholders. Qualitative themes were examined in relation to quantitative patterns to identify convergences, divergences, and complementarities. This narrative integration strengthened the explanatory power of the study and supported the assessment of inclusiveness in TEIs.

**Table 4.** Example of the thematic analysis process, with generating codes, identifying themes and subthemes, and supporting the data with quotations from the interviews.

Theme	Subthemes	Initial Codes	Exemplary Quotation
Strengthening global health partnerships through TEIs by fostering inclusiveness, trust, and credibility	Advancing global standing and investment through inclusivity	EU as guarantee and supporter to increase likelihood for the private sector to invest.	*‘I think that (soft power dynamics between actors) leads to improving access, actually, because most of it, I think, is acknowledged that there’s power dynamics exist, but for the greater part, they are actually working to improve access to medicines as we are benefiting from each other’s expertise and perspective’ - Interview 21, Private Sector Company*
		Key for ownership	
		Benefiting of EUs network and knowledge	
		EU as a supporter for increasing competitivity of a country by being present on the ground	
	Building trust for sustainable partnerships	Bringing together European Assistance and African countries to deliver a package.	*‘The advantage definitely is to show the giant European response and maybe in some cases approach so. So, you have a chance to bring together not only the assistance from the European Union, but also from the partner countries. And as such, you’re offering a kind of package, for the partners.’- Interview 3, European Member State Development Agency*
		Alignment of the European actors in regular meetings with partners.	
		More exchange and reachability of partners on the ground.	

## Results

### Member State inclusion and geographical distribution of partners in Team Europe Initiatives

The number of TEIs has expanded to 161, comprising 125 country-level, 32 regional, and four global initiatives. Of these, 26 focused on health as of 2025, based on data from the EU Joint Programming Tracker [17]. Initially, health-related TEIs were primarily implemented at country level, targeting specific national health challenges and engaging mainly with in-country stakeholders. However, the mapping conducted in this study indicates a shift towards an increasing number of regional initiatives. These regional TEIs engage a broader range of partners across countries, and demonstrate higher levels of interconnection, including linkages with TEIs from other thematic areas [17].

**Figure 1.**
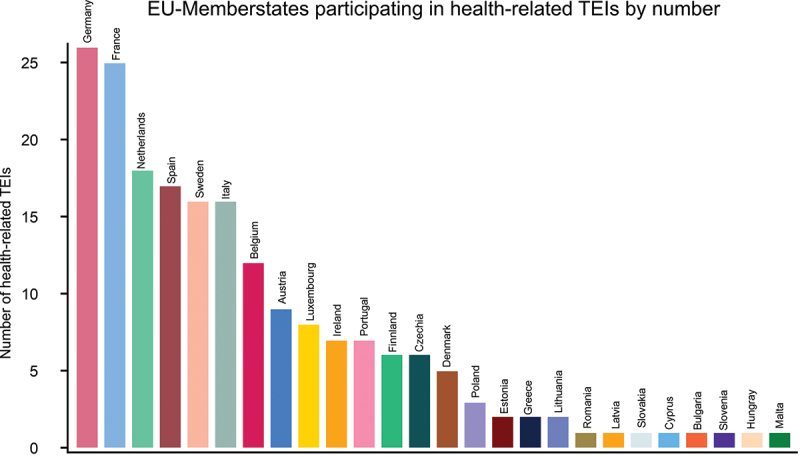
Member state participation (*n* = 26) in health-related TEIs by country, including all continents. *n* = number of all Member states of the European Union participating in at least one health related TEI.

The quantitative analysis also reveals a growing diversity in EU MS participation. By 2024, 26 MS, excluding Croatia, were involved in at least one health-related TEI. Germany and France were the most active participants, while other MS showed varying levels of engagement (Figure 1). Smaller MS have increased their participation over time, despite traditionally facing administrative and resource constraints. For example, countries such as Romania and Slovenia have engaged in initiatives beyond their previous geographic focus, including participation in the EU-Latin America and Caribbean Digital Alliance.

**Figure 2.**
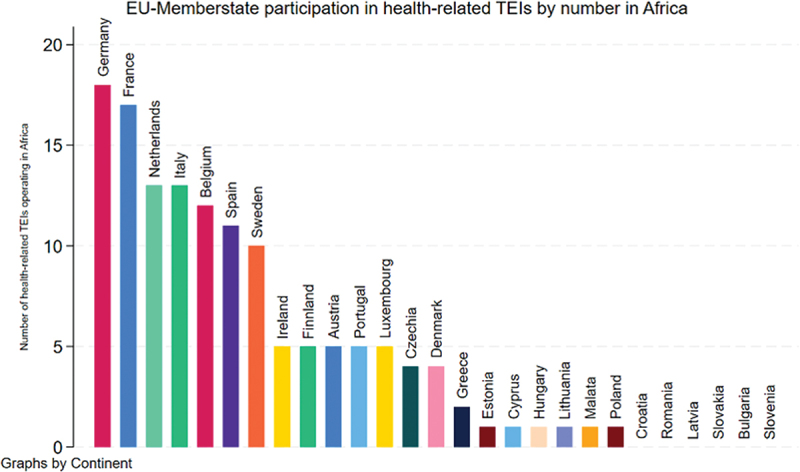
Member state participation in health-related TEIs in Africa (*n* = 21), including all 27 MS of the European Union. *n* = number of all TEIs with a focus on health, in Africa.

In Africa, which hosts the largest number of health-related TEIs, participation patterns largely mirror global trends. Germany and France remain the most prominent actors, followed by countries such as the Netherlands, Italy, Belgium, and Spain. In contrast, MS such as Poland or Estonia are less represented in Africa relative to their global engagement. At the time of data collection, MS, including Croatia, Romania, Latvia, Slovakia, Bulgaria, and Slovenia, were not involved in any health-related TEIs on the continent (Figure 2).

**Figure 3.**
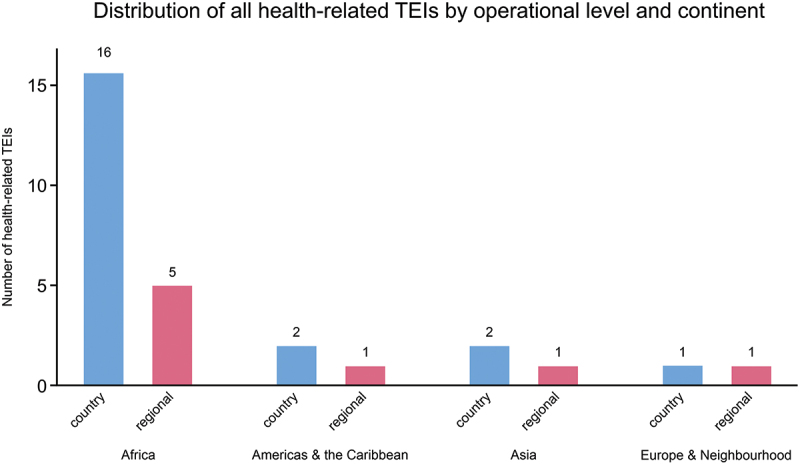
Distribution of health-related TEIs (*n* = 29) by continent and operational level (i.e. regional or country-level). *n* = number of all TEIs with a focus on health, globally.

Africa accounts for the majority of health-related TEIs (*n* = 21), reflecting both the scale of health challenges and longstanding EU engagement in the region. While most initiatives continue to operate at the country level, Africa also has the highest number of regional TEIs (*n* = 5), compared to an average of one per continent globally (Figure 3). This highlights a notable shift towards regional approaches addressing cross-border health challenges.

### Results of the thematic analysis of perceptions of health-related Team Europe Initiatives by actors involved at all levels

**Figure 4.**
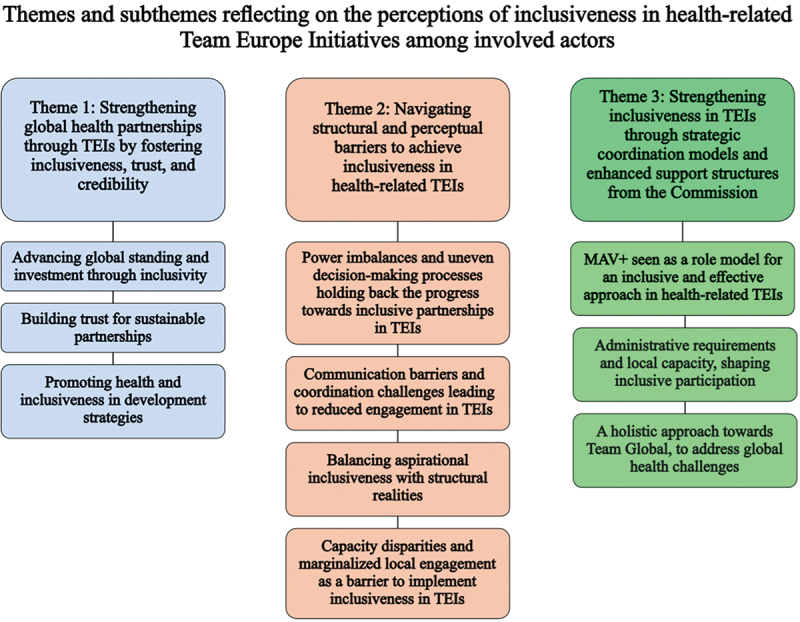
Themes and subthemes reflecting the perceptions of inclusiveness in health-related Team Europe Initiatives in Africa of actors involved.

The thematic analysis of 23 semi-structured interviews yielded three main themes with subthemes (Figure 4).

### Theme 1: strengthening partnerships in health through Team Europe Initiatives by fostering inclusiveness, trust, and credibility

This theme highlights the advantages of TEIs in promoting inclusivity, enhancing credibility, and promoting trust among partners in health-related development cooperation. TEIs were seen as a powerful tool to elevate the global profiles of both the EU and African partners.

#### Advancing global standing and investment through inclusiveness

TEIs were perceived to strengthen global standing and attract investment by including local partners. European respondents emphasized TEIs’ role in increasing EU visibility, while African partners highlighted opportunities to enhance their international positioning. African partners, including those from local authorities and private sector institutions, noted that TEIs enhance reliability, stability, and investor confidence. A national investment promotion authority highlighted:

*“TEIs enable the private sector to thrive, giving them confidence and legal certainty … you can address critical challenges of Africans from Africans instead of relying on the developed world.” - Interview 8, African Governmental Institution*

Smaller EU MS and local African partners emphasized that TEIs provide a stronger voice in global development and sustainable investment opportunities (Interviews: 14, 15, 16, 18, 21, 23). Other respondents noted that participation in TEIs positions health as a strategic priority across sectors (Interviews: 1, 10, 17, 19, 20).

#### Building trust for sustainable partnerships

Trust emerged as essential for sustainable TEI partnerships, especially for actors on the ground in Africa. Participants stressed the need to involve research institutions, private sector, academic institutions, and NGOs early, fostering long-term collaboration. Academic collaborations were highlighted as effective in promoting mutual respect and shared goals.

*“I know the European Union Commission has its own standard protocols on how to use their funds, established, but to benefit more, still there is a room for improvement, to hear what we want, and how you want it to be implemented so that it can impact more. So far, it is working, but it can be improved.”- Interview 23, African Governmental Institution*

Transparent communication was seen as crucial for aligning expectations. European respondents noted how shifting EU priorities or external influences, such as US elections, can undermine transparency. African partners cited the COVID-19 pandemic as a missed opportunity to strengthen trust and collaboration.

*“And, for instance, (…) like the Gates Foundation and others influencing largely the discussion, not just the global landscape, but even the World Health Assembly, and WHO. So yes, these, these power games, lead to fragmentation, and lead to a biased approach to certain health priorities also in the TEIs.”- Interview 19, European Commission Institution*

#### Promoting health and inclusivity in development strategies

All interviewees perceived TEIs as tools to prioritize health and foster inclusivity. TEIs create spaces for dialogue across regional and national organizations, enabling health initiatives to align with strategy plans and be implemented inclusively (Interviews: 1, 5, 23). TEIs were seen as beneficial for smaller development providers and underrepresented nations, enhancing engagement in global health discussions. EU presence on the ground was perceived as improving the understanding of local needs and boosting credibility. African local authorities and private sector actors emphasized that TEIs encourage collaboration, reflect diverse perspectives, reduce competition, and amplify Africa’s role in global health leadership (Interviews: 14, 21, 23).

### Theme 2: navigating structural and perceptual barriers to achieve inclusiveness in health-related TEIs

This theme addresses systemic challenges hindering TEIs’ full potential for working better together with partners in health. Structural, operational, and perceptual barriers create tension between aspirational goals and practical implementation, shaped by resource constraints, power imbalances, and differing priorities.

#### Power imbalances and uneven decision-making processes holding back the progress towards inclusive partnerships in TEIs

A recurring issue, brought up from smaller development agencies in Europe, was the unequal distribution of influence within TEIs, with larger EU MS like Germany and France often dominating decision-making processes. This dynamic limited the ability of smaller MS and African partners to shape priorities and assert their interests.

Historical linkages and long-established networks further reinforced these imbalances, creating a cycle where certain actors consistently held greater say in decision-making. This dynamic was seen in some scenarios, such as the development process of a TEI, as hindering the ability of less well-connected, though equally willing, partners to take a more meaningful leadership role in a TEI from the beginning. This imbalance of power was particularly evident in the prioritization of health security, a concept that elicited divergent interpretations. European and African participants even expressed contrasting views. Many European respondents criticized the focus on health security as a protectionist measure, which they saw as safeguarding the EU’s borders from epidemics and other health threats while neglecting local needs in partner regions. Conversely, African stakeholders viewed health security more positively, considering it a pathway to capacity building and achieving greater independence from external aid.

#### Balancing aspirational inclusiveness with structural realities

Resource constraints, staffing challenges, and bureaucratic demands often limited inclusiveness. Initiatives like MAV+ were praised for robust resource allocation and early local engagement. Many projects struggle due to fragmented funding and heavy reporting requirements. One respondent noted:

*“And each of them (funders) has a format for the reporting, et cetera. And this amount of work is often not considered as something that needs to be funded. But when you have a lot of funds, it is something that is a key issue.” - Interview 17, European Private Sector Company working in Africa*

COVID-19 exacerbated challenges by prioritizing urgency over early local engagement (Interviews: 2, 3, 10, 11, 13, 14, 19, 21, 22, 23). Addressing these structural issues was deemed essential to build trust and local ownership.

#### Communication barriers and coordination challenges lead to reduced inclusivity in TEIs

Communication challenges, such as language differences, time zones and frequent meetings, were seen to complicate collaboration. Smaller organizations with limited staff capacity struggle to engage meaningfully in discussions dominated by larger entities. Simplifying communication processes was seen as essential to fostering more inclusive partnerships.

#### Capacity disparities and marginalized local engagement as a barrier to implementing inclusiveness in TEIs

Imbalances in institutional and staff capacities constrained inclusiveness. Smaller organizations often could not participate fully in decision-making or monitoring. Well-established institutions were better equipped to navigate EU workflows and contribute effectively. Stable national health strategies further enabled inclusive participation by aligning initiatives with government priorities and fostering ownership.

Rwanda’s Health Sector Strategic Plan (HSSP IV, 2018–2024) was cited as a model of alignment, emphasizing community-based care and strong governance. It provided a solid foundation for TEI implementation by clarifying priorities, involving partners early, and facilitating coordination (Interviews: 5, 8, 23).

### Theme 3: strengthening inclusiveness in Team Europe Initiatives through strategic coordination models and enhanced support structures from the Commission for EU Member states and local partners

This theme synthesizes the key insights from the experiences with the TEI ‘MAV+’ and broader ideas for improved support and perspective shifts in TEIs.

#### MAV+ is seen as a role model for an inclusive and practical approach to health-related TEIs

From all interviews, it emerged that the TEI MAV+ demonstrates best practices in inclusiveness, synergy, and accountability. A participant highlighted:

*“On MAV+, that really is, I think, also a platform of shaping things, of comparing things, and of doing things in a coordinated manner.” - Interview 12, European Member State Agency*

Seen as a success from the European perspective, this TEI combines a top-down and bottom-up approach, which allows for early local engagement, as well as the fostering of trust and mutual growth. Furthermore, African partners from the private sector on the ground emphasized that MAV+ illustrates how strong EU representation enhances credibility and commitment to equal partnerships. Despite MAV+‘s perceived success, broader challenges remain, according to the interviewees. Partners from Africa, as well as respondents working for an EU delegation in an African country, called for the European Commission to reallocate resources from Brussels to EU representatives in the field in Africa and to simplify bureaucratic processes to enable better local participation. However, high-ranking EU representatives in Africa were seen by other stakeholders as key to enhancing project visibility and credibility:

*“It is an example of working towards equal partnerships. (…) and now, with such a high representation from the EU, I think that’s the right message to send to our partners.”*- *Interview 1, International Organization*

#### Administrative requirements and local capacity, shaping inclusive participation

Interviewees across European and African institutions, international organizations, and the private sector highlighted that administrative and reporting requirements associated with EU-funded initiatives can affect partners’ ability to participate effectively in TEIs (Interviews: 1, 4, 5, 7, 8, 14, 16, 17). EU funding mechanisms were described as involving complex written documentation and formal reporting procedures, which contrast with the often informal, rapid, and verbal communication practices used in day-to-day collaboration (Interviews: 1, 4, 5, 7, 16, 17). A representative from a development financing institution explained this disconnect:

*“In many cases, communication between partners on the ground is quite direct and often happens through informal or verbal exchanges. However, the reporting requirements for funders are very detailed and written, which means that this information has to be translated into formal documentation later on.”*- *Interview 4, European Member State Agency*

These requirements were described as challenging not only for African partners but also for organizations with limited experience managing EU-funded projects (Interviews: 13, 14, 15, 17, 18, 20, 21). While designed to ensure accountability and transparency, they can create significant administrative burdens, particularly for actors without dedicated staff or institutional structures for grant management. In contrast, many European institutions benefit from specialized administrative units for proposal writing, project management, and reporting. A private sector respondent emphasized the need for capacity-building:

*There is a need to improve the capacity of local partners so they can better understand what funders expect from financial reports and project documentation.*

*Interview 17, European Private Sector Company working in Africa*

Overall, these challenges reflect broader structural features of EU funding systems rather than region-specific constraints. Interviewees consistently highlighted the importance of simplifying procedures and strengthening capacity to enable more equitable participation. Tools such as the Operational System (OPSYS) were identified as useful in improving transparency and coordination across partners (Interviews: 1, 8, 10, 13, 18, 21).

#### A holistic approach towards ‘Team global’ to address global health challenges and power imbalances in TEIs

Both perceptions about MAV+ and the broader reflections on TEIs highlight the need for a global, more holistic approach to health challenges. By fostering mutual learning and collaboration, respondents felt that TEIs can address systemic barriers and promote sustainable solutions for global health. Participants from both European and African sides emphasized that TEIs should extend beyond addressing immediate regional needs to establish systems that enable partners to learn from one another’s strengths and weaknesses. One participant from Africa emphasized this need for a shift by calling it Team Global, not Team Europe (Interview 23).

## Discussion

### Geographic distribution of TEIs

A notable finding is the geographic distribution of health-related TEIs. Despite a diverse set of initiatives, there is a persistent focus on African countries (Figure 3), while regions such as Latin America, Asia, and the Middle East receive less attention. This reflects the EU’s engagement with Africa, shaped by historical ties, shared development priorities, and a high disease burden alongside a more fragile health system. Recent political commitments, such as the ‘Joint Vision for 2030’ at the 6th EU-AU Summit, support this focus [24,25]. The COVID-19 pandemic highlighted this interdependency, regarding vaccine equity and Africa’s aspirations for local vaccine production, which now feature prominently in the EU’s health commitments [26]. While TEIs emphasize inclusive partnerships and local engagement, they operate within a system influenced by EU MS political and economic interests. Initiatives like MAV+ aim to improve vaccine equity, yet they remain shaped by structural constraints and broader global health politics. TEIs’ objectives should therefore be interpreted alongside these contextual factors, as inclusiveness and equity are negotiated within a system where northern partners often hold considerable power. The concentration of TEIs in Africa aligns with urgent health challenges. However, geographic inclusiveness should be understood as equity-driven allocation rather than equal distribution. Prioritizing Africa allows for intensive engagement where disease burdens are higher while recognizing that other regions, such as Latin America, face rising health challenges but receive comparatively less attention [27]. Despite the rationale for focusing on Africa, this uneven distribution may unintentionally limit the EU’s broader global health ambitions by overlooking other vulnerable regions and missing opportunities for cross-regional learning. Expanding the geographic scope of TEIs and fostering interconnections across countries or continents would better reflect the complex and varied health needs globally. Research underscores this finding of diversification being essential to foster equitable partnerships and avoid regional blind spots in global health initiatives [27].

### Uneven Member state participation in health TEIs

The study reveals disparities in participation among MS. Larger MS such as Germany and France are the most active contributors to TEI funding and leadership. However, simple counts of participation may misrepresent involvement if economic capacity is not considered. Larger MS generally have greater resources and larger official development assistance (ODA) budgets relative to smaller MS. For example, Germany consistently ranks among the highest donors in absolute ODA and as contributors to EU development cooperation instruments [28]. In contrast, smaller MS, with lower GDP and smaller ODA allocations, have constrained fiscal and administrative capacity, limiting their ability to engage in externally funded initiatives.

Equity in global health cooperation, therefore, does not imply equal numerical participation but proportional engagement relative to capacity. Economic indicators such as GDP and ODA volumes are routinely used in comparative analyses to contextualize a state’s ability to commit resources [29,30]. Aid allocation and donor engagement are shaped by political and strategic considerations [31]. From this perspective, the prominence of larger MS in TEIs reflects structural realities of development finance rather than a lack of willingness from smaller MS. Distinguishing formal participation from substantive capacity is crucial when assessing inclusiveness in global health partnerships. These patterns are not unique to TEIs but reflect broader European development cooperation trends, as noted by Buse and Hawkes [32] and studies on EU foreign aid dynamics [33,34]. Smaller MS struggle to participate due to limited staff capacity and political will. Additionally, TEIs are frequently perceived as driven by the European Commission, concentrating on responsibilities and decision-making among larger actors. This centralization can sideline smaller states, limiting diversity of voices and contributions within TEIs. Broad engagement of MS is essential for fostering inclusive partnerships, enriching perspectives, and equitably sharing responsibilities and benefits across the EU [13,14,35].

To address these disparities, the study suggests prioritizing capacity-building for smaller MS to enhance meaningful participation. Mechanisms such as pooled funding, streamlined application processes, and targeted technical support could encourage broader engagement both within Europe and globally. Such measures would enhance inclusiveness and distribute the burdens and benefits of global health collaboration more equitably across EU MS, ensuring that smaller states can contribute effectively alongside larger counterparts.

### Regional versus country-level TEIs: a shift towards greater inclusivity?

A significant finding of this study is the distribution of health-related TEIs between country-specific and regional initiatives. Africa stands out with the highest proportion (63%) of regional TEIs compared to any other continent (Figure 3). Interviewees noted that regional TEIs can facilitate coordination across borders and multi-stakeholder engagement, bringing together ministries of health, local authorities, civil society, and EU delegations from multiple countries to align priorities and pool resources. However, the findings also indicate that regional TEIs do not automatically guarantee context-sensitive or equitable participation. Involving multiple countries can dilute individual partners’ influence and complicate decision-making, especially when capacities vary or national interests diverge, making inclusiveness dependent on implementation, engagement, and governance structures.

Regional TEIs are particularly valuable in addressing health issues spanning multiple countries, as they promote more inclusive and context-sensitive approaches. By incorporating diverse national contexts and engaging in a broader range of stakeholders, they enhance flexibility and coordination. Studies of regional health cooperation in Africa corroborate that cross-border mechanisms can facilitate joint planning, pooled procurement, and harmonized surveillance, although outcomes vary based on institutional strength and political commitment [36,37].

The emphasis on regional TEIs in Africa reflects broader international trends in regional health governance, exemplified by WHO frameworks advocating integrated, cooperative approaches to health system strengthening [38]. Such regional coordination aligns with SDGs and principles of health system resilience and equity [35,39]. In contrast, TEIs in other continents are predominantly country-level, allowing deeper alignment with national priorities and clearer accountability structures. Africa’s higher proportion of regional TEIs reflects shared health challenges, including infectious diseases and health system weaknesses, which necessitate coordinated responses. Pre-existing AU-EU partnerships also support regional networks for collaboration. The EU’s focus on regional TEIs in Africa can thus promote greater inclusivity by engaging multiple stakeholders and avoiding interventions confined to national silos, in line with the EU Global Health Strategy 2022 [10]. As Kickbusch and Cassar Szabo (2014) emphasize, regional collaboration in health not only improves access to resources but also strengthens local ownership and builds long-term capacity, key elements for ensuring the effectiveness and inclusivity of health interventions [27]. From a power and decision-making dimension, regional TEIs represent a shift toward decentralized, multi-stakeholder governance, distributing decision-making authority across countries and actors. The EU’s partnerships with the AU help facilitate this shared governance, reducing top-down control and fostering more balanced influence. This aligns with broader trends in global health governance that emphasize collaborative, polycentric approaches rather than hierarchical models [40].

In contrast, other continents tend to have more country-level TEIs, which may limit regional cooperation and reinforce centralized power structures. Expanding regional TEIs beyond Africa could further democratize health governance and align with the EU’s goals of inclusive, coordinated responses to global health challenges. However, to ensure that such expansion truly enhances inclusiveness, mechanisms that safeguard equitable participation and voice for smaller MS and local partners are essential, preventing regional modalities from reproducing existing power asymmetries.

### Enhancing inclusiveness and local ownership in Team Europe Initiatives

Local partners, including national governments, CSOs, and health institutions, are critical to shaping TEIs to align with national priorities and community needs. Their involvement enhances programmatic relevance, strengthens local capacity, and supports long-term sustainability [41]. However, participation of local actors remains inconsistent due to structural barriers such as limited financial resources, institutional capacity, and bureaucratic complexities in EU funding mechanisms. Local ownership is a key determinant of TEI success, strengthening both legitimacy and impact. Initiatives involving local actors in design and implementation are more likely to be embraced by communities and integrated into national health strategies [42]. Conversely, externally driven interventions that do not align with national priorities may face resistance and struggle to achieve lasting impact. Ensuring local ownership also aligns with global aid effectiveness commitments, such as the Paris Declaration, which emphasizes aligning international support with national strategies [43]. Importantly, the findings of this study suggest that regional and country-level approaches should not be understood as competing models, but as complementary governance levels. While regional TEIs can facilitate coordination across countries and address cross-border health challenges, meaningful inclusiveness and ownership ultimately depend on how well these initiatives are anchored in national systems and priorities. Interviewees highlighted that even within regional initiatives, the extent to which national actors are meaningfully involved in agenda-setting and implementation varies considerably. Without strong national engagement, regional approaches risk reproducing top-down dynamics rather than enhancing inclusiveness. To address these challenges and ensure the continued success of health-related TEIs, the EU must take deliberate steps to enhance the link between regional coordination and national ownership. This includes enhancing participation mechanisms and promoting greater transparency in the allocation of resources. Additionally, the EU should adopt more flexible funding and governance structures that facilitate direct collaboration with local actors. Simplifying administrative requirements for CSOs and health institutions in low-resource settings, strengthening coordination mechanisms between EU institutions and local governments, and ensuring that funding is equitably distributed will be key to fostering inclusiveness. Furthermore, the findings suggest that TEIs should promote more multi-level participatory governance structures, where local actors contribute not only as implementers but also as co-creators of policies and programs. Aligning with recent calls in global health for reforms that emphasize country ownership, self-reliance, and synergy across partners. The Gavi Leap initiative, as articulated by Nishtar (2025), advocates a radical transformation of global health institutions toward a country-centric, simplified, and partnership-oriented architecture that better reflects local priorities and reduces burdensome centralized processes, a direction that resonates with the interviewees view of a stronger participatory governance in TEIs [44]. In this context, regional TEIs can support coordination and shared learning, but their effectiveness depends on ensuring that decision-making authority and ownership remain grounded at the country level.

### Building trust and transparency for sustainable partnerships between affected actors

Trust emerged as a cornerstone of effective collaboration in relation to the TEIs, emphasizing its importance in building lasting, successful partnerships. The study supports the idea that trust is vital not only between political leaders but also among academic institutions, researchers, and grassroots actors, ensuring equitable and effective partnerships.

Transparency in communication was also highlighted as essential for fostering trust. Clear, open communication reduces misunderstandings and ensures that all parties, regardless of their size or power, can contribute meaningfully to decision-making processes. This aligns with the broader literature on transparency in international health partnerships, which emphasizes its role in mitigating power imbalances and enhancing accountability [45].

#### Limitations and strengths

The quantitative analysis relied on publicly available documentation, limiting coverage of all EU-AU cooperation and informal partners. The qualitative component partly addressed this gap, though selection bias remains due to purposive and snowball sampling. A potential Europe-centric bias was mitigated by including European and African stakeholders, but perspectives from underrepresented local partners remain limited due to capacity and access constraints. Triangulation of quantitative and qualitative data broadened perspectives and revealed gaps between formal participation and experienced inclusiveness. As all authors are Europe-based, positionality may have influenced design and interpretation. To mitigate this, diverse stakeholders from European and African contexts were included, and the study design was informed by literature and practitioner input.

## Conclusion

TEIs transitioned from a pandemic response mechanism into a cornerstone of the EU’s global health and development strategy. This study reflects on TEIs possible promote cross-border coordination, multi-stakeholder engagement, and pooling resources, yet their inclusiveness and effectiveness depend on governance, partner engagement, and alignment with national priorities.

While TEIs are perceived as promising instruments to foster inclusiveness in global health cooperation, strengthening trust, expanding partnerships, and potentially reducing African dependency on development aid, important challenges remain. Insufficient involvement of local partners in decision-making processes has led to persistent power imbalances and limited agency, with local actors often dependent on government mediation to participate meaningfully. Furthermore, the scope of collaboration remains narrowly focused on European governmental and institutional actors, underscoring the need to broaden the ‘Team’ to include a wider array of stakeholders.

To realize the potential of TEIs as inclusive, effective, and sustainable mechanisms, the EU must address these limitations by enhancing local ownership, diversifying partnerships, and fostering equitable governance structures. Doing so will not only reinforce the EU’s role as a trusted global health leader but also contribute to building more resilient health systems founded on mutual collaboration and shared responsibility.

## Supplementary Material

Supplementary material.docx

GRAMMS.docx

## Data Availability

The data cannot be shared publicly due to the privacy of participants of the study. The data will be shared on reasonable requests to the corresponding author.
