# Supplementary material for: “Assessing inclusiveness in Team Europe Initiatives: a mixed-methods study of EU-Africa health cooperation”
Source: Glob Health Action. 2026 Jun 3;19(1):2680837. doi: 10.1080/16549716.2026.2680837 (PMC13235253; doi:10.1080/16549716.2026.2680837)
Supplement: Supplementary material.docx [file ZGHA_A_2680837_SM4808.docx]

Supplementary material

Appendix1: Interview guide.

| **Interview Guide**  **Topic 1: Roles in the TEIs**   1. Tell me about your role in the TEIs? 2. What are the main partners you are collaborating with?  - How and on what stage were these actors involved?  1. What are your thoughts on if there are countries/actors over-/or underrepresented in the TEIs?  - What do you think are the barriers for underrepresented countries/actors?  1. Could you reflect on the power distributions among involved actors?  - What do you think this leads to?   **Topic 2: Inclusive Partnership and local ownership**   1. What are your perceptions on the involvement of local partners?  - How were consultations conducted with local partners?   How do you perceive the impact of the local actors and local development agendas in the TEIs? |
| --- |

| 1. What do you think are the advantages/disadvantages of the governance structure of the TEIs, in comparison to the traditional development cooperation? (Joint programming)  - Please reflect on the terms:  1. Europeanness in the TEIs (Inclusiveness of all Member states) 2. Transformation for partner countries (Are needs and priorities met)  - How do you think they are implemented in the TEIs?  1. How do you feel the concept of the TEIs encourages active participation of EU- and non-Eu-actors (such as local DFIs and banks, private sector, civil society, and local authorities?)  - What do you think is necessary to improve the participation of various stakeholders towards a successful implementation of the TEIs?  1. Which aspects do you think are essential for a future successful implementation phase of the TEIs in terms of partnership? 2. Any additional thoughts you would like to share? |
| --- |

**Appendix 1: Interview Guide.**
