# Supplementary material for: “Assessing inclusiveness in Team Europe Initiatives: a mixed-methods study of EU-Africa health cooperation”
Source: Glob Health Action. 2026 Jun 3;19(1):2680837. doi: 10.1080/16549716.2026.2680837 (PMC13235253; doi:10.1080/16549716.2026.2680837)
Supplement: GRAMMS.docx [file ZGHA_A_2680837_SM4807.docx]

**Good Reporting of A Mixed Methods Study (GRAMMS) checklist**

| **Guideline** | **Section: page (line) in the manuscript** |
| --- | --- |
| Describe the justification for using a mixed methods approach to the research question | Methods: p.7 (line 102-122) |
| Describe the design in terms of the purpose, priority and sequence of methods | Methods: p.8 (line 123- 126) |
| Describe each method in terms of sampling, data collection and analysis | Method: pp.8-9 (line 123- 150) |
| Describe where integration has occurred, how it has occurred and who has participated in it | Method: pp. 8-9 (line 138-141) and 153-165) |
| Describe any limitation of one method associated with the present of the other method | Discussion: p.25 (line 511-520) |
| Describe any insights gained from mixing or integrating methods | Discussion: p.25 (line 516-520) |
